# Using data-driven sublanguage pattern mining to induce knowledge models: application in medical image reports knowledge representation

**DOI:** 10.1186/s12911-018-0645-3

**Published:** 2018-07-06

**Authors:** Yiqing Zhao, Nooshin J. Fesharaki, Hongfang Liu, Jake Luo

**Affiliations:** 1 Department of Health Informatics and Administration, Center for Biomedical Data and Language Processing, University of Wisconsin-Milwaukee, 2025 E Newport Ave, NWQ-B Room 6469, Milwaukee, WI 53211 USA; 2 Division of Biomedical Statistics and Informatics, Mayo Clinic, Rochester, 205 3rd Ave SW, Rochester, MN 55905 USA

**Keywords:** Knowledge modeling, Sublanguage analysis, Natural language processing, Semantic network, Big data analysis, Medical imaging, Text mining, Information extraction

## Abstract

**Background:**

The use of knowledge models facilitates information retrieval, knowledge base development, and therefore supports new knowledge discovery that ultimately enables decision support applications. Most existing works have employed machine learning techniques to construct a knowledge base. However, they often suffer from low precision in extracting entity and relationships. In this paper, we described a data-driven sublanguage pattern mining method that can be used to create a knowledge model. We combined natural language processing (NLP) and semantic network analysis in our model generation pipeline.

**Methods:**

As a use case of our pipeline, we utilized data from an open source imaging case repository, *Radiopaedia.org*, to generate a knowledge model that represents the contents of medical imaging reports. We extracted entities and relationships using the Stanford part-of-speech parser and the “Subject:Relationship:Object” syntactic data schema. The identified noun phrases were tagged with the Unified Medical Language System (UMLS) semantic types. An evaluation was done on a dataset comprised of 83 image notes from four data sources.

**Results:**

A semantic type network was built based on the co-occurrence of 135 UMLS semantic types in 23,410 medical image reports. By regrouping the semantic types and generalizing the semantic network, we created a knowledge model that contains 14 semantic categories. Our knowledge model was able to cover 98% of the content in the evaluation corpus and revealed 97% of the relationships. Machine annotation achieved a precision of 87%, recall of 79%, and F-score of 82%.

**Conclusion:**

The results indicated that our pipeline was able to produce a comprehensive content-based knowledge model that could represent context from various sources in the same domain.

**Electronic supplementary material:**

The online version of this article (10.1186/s12911-018-0645-3) contains supplementary material, which is available to authorized users.

## Background

A *knowledge model* is a formalized representation of information in a given domain. The graphical representation of a knowledge model consists of semantic categories as nodes and semantic relationships as edges. A knowledge model can be employed in order to transform unstructured text data into a computable logical format. For example, Weng et al. developed EliXR, a model for formalizing clinical research eligibility criteria [1]. In this model, a frame-based (based on pre-defined event frame e.g. drug exposure + frequency + dosage) and ontology-dependent template (e.g. extract drug name using ontology) were used to extract information into 20 clinically relevant semantic types (e.g., medication, dosage) from eligibility criteria. The knowledge model was able to cover a 99.8% of the content with average labeling error rate of 5.9%. Bashyam et al. developed a system that provided an overview of the patient’s imaging data in a model with four dimensions: time, space, existence, and causality [2]. In a similar manner, Coden et al. proposed a Cancer Disease Knowledge Representation Model (CDKRM), which was able to automatically extract information from free-text pathology reports [3] by incorporating Natural Language Processing (NLP), machine learning, and domain-specific rules. In general, the described knowledge models significantly facilitate the process of retrieving information through structuring the free-text medical documents.

Furthermore, recent studies have shown a great potential for using knowledge model components as machine learning features. To clarify, we mentioned this to demonstrate the significance of generating a knowledge model (the end product of our work). But our method doesn’t involve any machine learning step. For example, Yetisgen-Yildiz et al. [4, 5] developed a pipeline to automatically extract semantic components from radiology reports. They first constructed a knowledge model (with an ontology of 11 section categories) of radiology reports sections to identify section boundaries using rule-based approach. Then features (both syntactic and semantic) for each section were extracted and fed into a classification algorithm in order to automatically identify critical clinical recommendations. The pipeline achieved an F-score of 0.75. In a study [6], thromboembolic diseases described in radiology reports were detected using NLP and machine learning techniques. In this study, NLP techniques were used to extract concepts of thromboembolic diagnosis and incidental findings, which were then employed as features of a supervised machine learning algorithm. The proposed conceptual model achieved performance improvement in all cases with F-score of 0.98, 1.00, and 0.80 for pulmonary embolism identification, deep-vein thrombosis, and incidental clinically relevant findings, respectively.

It has been also shown that the knowledge model plays a significant role in setting up a knowledge base when the text mining techniques are used [7–9]. Moreover, with the growing need for integration of data sources (e.g. written protocol, EHR data, published case report) in order to establish a comprehensive knowledge base, a domain-specific knowledge model becomes essential for uniform content representation. In addition, the importance of knowledge model as a fundamental component of developing clinical decision support systems has been studied previously [10, 11]. Some existing efforts that address this need include: 1) setting up a Common Data Model (CDM) or the use of Resource Description Framework (RDF) to represent elements and relationships in a text [10, 12–14]. 2) using ontologies as knowledge models to build automatic information retrieval systems [8, 15–17]. However, building automatic information retrieval systems based on CDMs is difficult since the automatic mapping of entities to those data models can be totally challenging, and thus, the current efforts usually involve a significant amount of manual labeling in the first step of developing a system [14, 18, 19]. On the other hand, although ontologies have been widely used for knowledge representation, their complex hierarchy and insufficient relations between concepts have restricted the potential of using them to mine the most clinically relevant knowledge automatically and precisely. Moreover, an ontology building is a time-consuming process – usually expert-based and heuristic [15, 20, 21].

To address the unmet need (for integration of data sources to establish a comprehensive knowledge base), we proposed a data-driven sublanguage pattern mining method to induce a context-based knowledge model. According to Zellig Harris’s sublanguage principle, restricted domains (e.g., biomedical) have unique syntactic patterns and a limited number of semantic types [22, 23]; therefore, the language grammar can be revealed and subsequently, the semantic relations can be identified as syntactic and/or semantic patterns. A combination of NLP and semantic network analysis techniques make it possible to reveal a sublanguage pattern model in a domain-specific content [24, 25]. In contrast to the above top-down approaches (CDM, RDF, ontology, etc), our method employed a novel, bottom-up, context-based approach that combines both syntactic analysis and data-driven semantic network analyses in order to explore semantic relations in a specific corpus. In this paper, we utilized an open source medical image report data repository as a use case. However, the pipeline can be extended to other domain-specific knowledge models as well.

## Methods

**Fig. 1:**
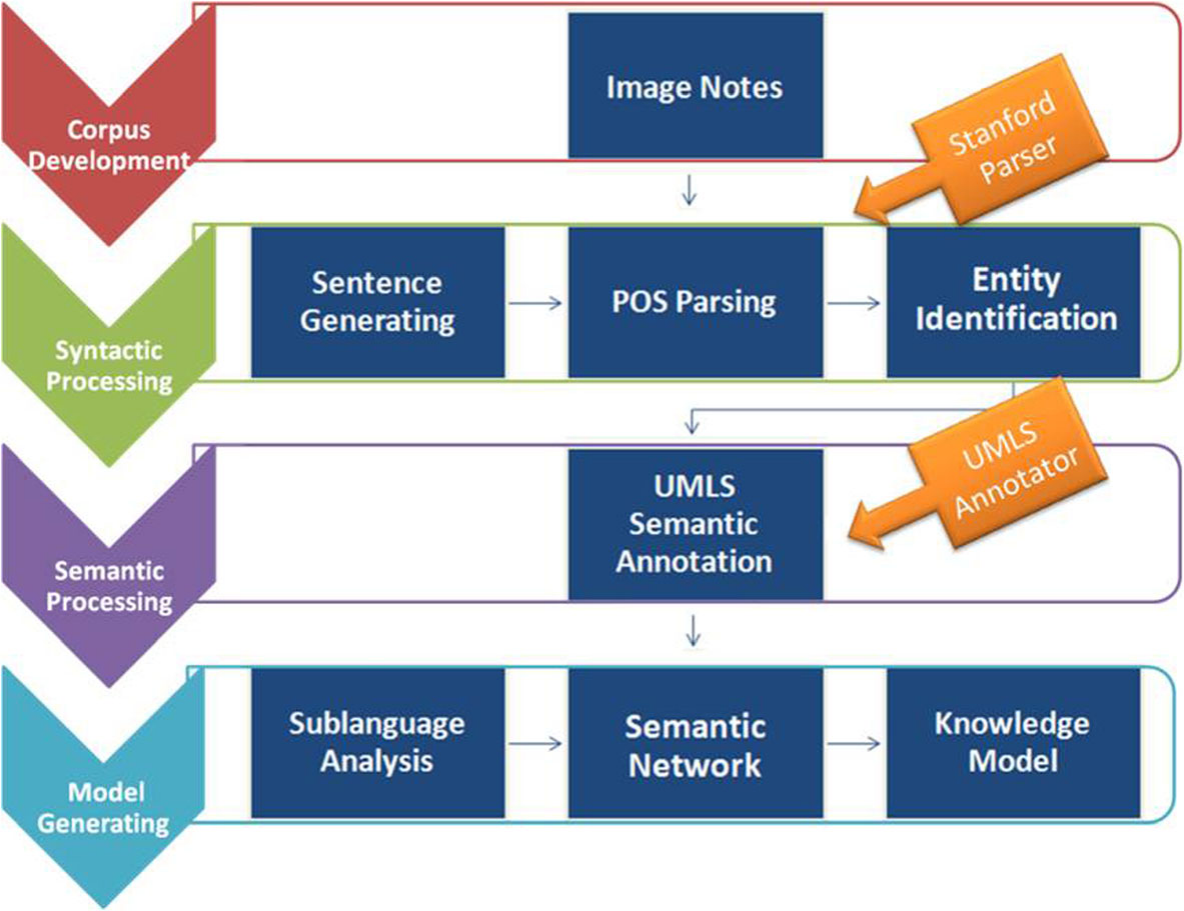
System pipeline: (1) Corpus development (using Jsoup), (2) Syntactic processing (using Stanford Parser), (3) Semantic processing (using UMLS Annotator), (4) Knowledge model generation

The proposed method has four major steps: corpus development, syntactic processing, semantic processing, and knowledge model generation. Functionally speaking, the entire pipeline is able to complete tasks of entity recognition, relation extraction, semantic category assignment and semantic network analysis. Sublanguage patterns were detected with semantic network analysis and a comprehensive knowledge model was then constructed based on the sublanguage patterns. Figure 1 shows the data-driven pipeline for knowledge model generation of the image reports.

### Corpus development

Radiopaedia.org [26] contains a large variety number of medical imaging case reports, along with physicians’ in-depth case analyses and discussions. The data covers cases in 19 different body systems (e.g., breast, cardiac, spine) with 14 different modalities (e.g., CT, MRI). Using data in Radiopaedia.org, we built a corpus by parsing (with JSoup Package [27]) the data consisting of textural notes of clinical images such as body system, user-defined keywords, patient demographics, image modalities, clinical findings, and case discussion. The extracted data contained 23,410 physician-remarked medical image reports as of Feb 7, 2017. The first case published on Radiopaedia.org was May 7, 2008. Thus, the collected corpus represents a wide range of contemporary radiology case reports with different modalities, age groups, ethnic groups and body systems.

### Syntactic processing

Sublanguage patterns can be revealed through identification of semantic relations based on language grammar. So, syntactic processing such as Hearst’s lexico-syntactic analysis is an important step of sublanguage pattern mining, which provides users with “is-a” relationships by extracting the hypernymic/hyponymic relations from the text [28, 29] despite diverse syntactic variations. However, this method has limited ability to reveal other relationships such as location, causality, and indication while these relationships are important in medical imaging domain [30, 31]. Representing sentences with predicate-argument structures (PAS) combined with shallow semantic parsing are usually used for more complicated patterns within a medical text [32, 33]. These methods always require annotated corpora for training supervised machine-learning systems; however, there are very limited annotated clinical narrative corpora within the clinical domain, many of which may not be easily adapted to the medical imaging domain. As a result, we extracted and examined “Subject:Relationship:Object” (SRO) structures [34, 35] from imaging notes to generate a semantic network and to formulate a knowledge model. SRO structures are considered the core units for representing the content of each note. We examined “Subject/Object” pairs in a process similar to Hearst’s examination of hypernymic/hyponymic pairs, but with more comprehensive relationships between entities.

We reorganized each imaging note into short sentence segments by period, comma, colon, “and”, “which”, and so on. Next, we used the Stanford part-of-speech (POS) parser version 3.5.2 [36, 37] to analyze the syntactic structure of each sentence to extract the “Subject:Relationship:Object” parsing schema. Given this schema, we first identified the verb phrases (VP) or prepositional phrases (PP) in each parse tree and then determined whether each phrase was an embedded structure. A parse tree [38] is an ordered, rooted tree that represents the syntactic structure of an English sentence according to some context-free grammar using grammatical tags for each word or phrase together with the relationships between words and phrases. An embedded structure is defined as a verb phrase or prepositional phrase that contains other VP or PP within its structure. We also extracted maximal (longest) noun phrases (NP) and adjective phrases (ADJP) as entities, and marked them as a Subject or Object. Adverbs were separately extracted as modifiers of either Subject, Relationship or Object in the “Subject:Relationship:Object” schema.

We generalized four types of embedded structures: (1) NP + VP:(Verb+VP:(Verb +NP)), such as “A has become B”. This structure usually relates to the passive voice or past tense. The verb is extracted as a combination of two words e.g., “have become”, so that we could keep the tense of relation in our schema. (2) NP + VP:(Verb +PP:(Prep +NP)), such as “A present with B”. In this structure, the main relation was extracted as the entire phrasal verbs “present with” in order to keep the phrasal verbs intact. (3) NP+ VP:(VB + VP:(Verb +PP:(Prep+NP)), such as “A is associated with B”. This structure is a combination of the first two. (4) NP + VP:(Verb +NP + PP:(Prep+NP)), such as “A demonstrated a patient with previous history”. This is a postpositive structure; the main relation was extracted only by using the verb, but the Object is considered to be the combination of NP + PP (in this case, “patient with previous history”). This is a postpositive structure, and the main relation is extracted only by using the verb, while the Object is a combination of NP and PP (in this case, “patient with previous history”). This is a novel step, as most previous studies only deal with simple syntactic patterns, but not the nested ones, which could lose embedded syntactic relations between words and phrases.

### Semantic annotation

After extracting the relationships between the medical imaging entities, we annotated each entity in the SRO structure with its semantic labels. In this paper, “entity” refers to semantically taggable phrases. We used the Unified Medical Language System (UMLS) and SRO as our semantic reference and labeling structure, respectively. The UMLS is a knowledge source that integrates biomedical concepts from various controlled vocabularies, classifications, and other biomedical ontologies [39]. This semantic labeling method is completely different from previous ones that were based on a set of manually defined event templates [40].

A UMLS semantic tagger was used to assign a semantic type to each NP or ADJP (entities). The details of the tagger have been described in [41]. While most previous methods tagged all nouns/adjectives in an identified noun phrase [42, 43], we assigned only one tag to each NP/ADJP by extracting the maximal one. The tag is defined to be the semantic type of the last UMLS-recognizable entity in an NP/ADJP. For example, our method assigned the semantic annotation of *Observation* for the whole phrase “right breast pain” instead of a list of three separate annotations -- *Location + Body Part + Observation.*

### Knowledge model generation

To reveal the sublanguage pattern, we summarized the semantic types occurring in the corpus and visualized entity relationships using a co-occurrence-based semantic network. Co-occurrence incidence is defined as two semantic types, the Subject and Object, respectively, in one relation. Based on the induced semantic network, we discovered the network concentrates primarily on the top 40 semantic types, indicating a strong sublanguage pattern in the radiology case report corpus. We selected top 40 semantic types because increasing the number of semantic types beyond 40 doesn’t improve entity coverage significantly (~ 98.1% if selected top 50) but will introduce complexity in the model significantly. Moreover, semantic types ranking 41 or beyond are typically not related to medical image domains and could have semantic type mapping errors.

**Fig. 2:**
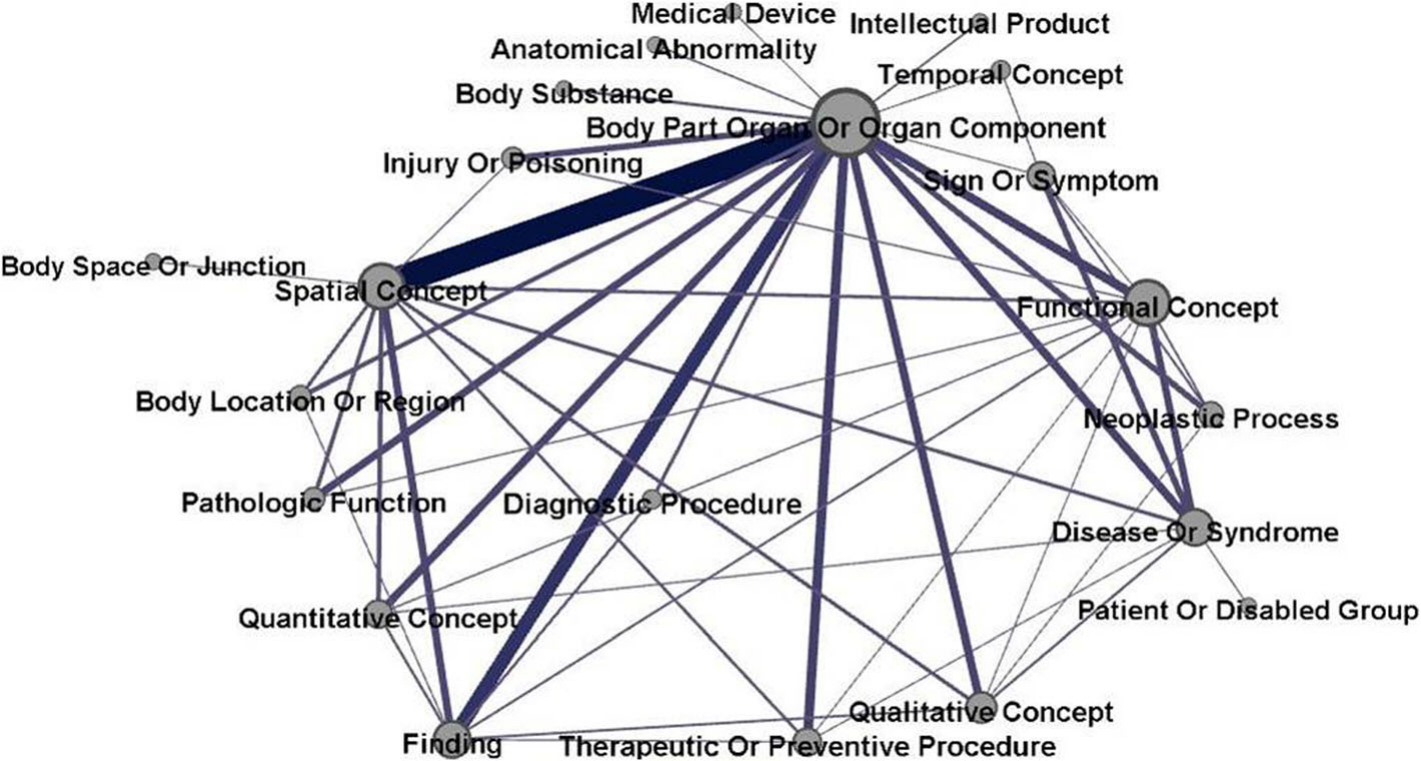
Co-occurrence network of top 40 semantic types (subgraph). The thickness of the edge demonstrates weight (the number of co-occurrence incidences); a thicker edge means more co-occurrence incidences exist in the relation. The size of the nodes indicates connectivity (the number of other nodes connected to it). The network graph represents the complexity of the semantic co-occurrence pattern of semantic types in imaging notes

We selected the top 40 semantic types that have the highest contents coverage (98% of overall UMLS-recognizable entities), which were further regrouped according to both the UMLS semantic hierarchy and the domain-specific semantic network (Fig. 2). We also added four conceptually important semantic types according to expert’s advice (despite its low frequency in our corpus; marked with “*” in Table 1). The rationale and results of semantic regrouping have been discussed in the Discussion section. A Semantic types are the original semantic labels defined in the UMLS system; the semantic categories defined in this study are then generated by regrouping semantic types. Finally, we formulated a knowledge model using nine induced semantic categories and five original semantic types (Table 1).

**Table 1:** Regrouping of UMLS semantic types to form 14 semantic categories (four conceptually important semantic types are marked with “*”)

New Semantic Category	Included UMLS Semantic Types	Type Counts
Abnormality	Anatomical Abnormality, Acquired Abnormality, Congenital Abnormality	3
Body Part	Body Part Organ or Organ Component, Body Substance, Body System*, Tissue, Cell, Gene or Genome, Receptor*	7
Classification	Classification	1
Functional Concept	Functional Concept	1
Location	Spatial Concept, Body Location or Region, Body Space or Junction	3
Medical Activity	Diagnostic Procedure, Therapeutic or Preventive Procedure, Laboratory Procedure, Health Care Activity, Research Activity, Activity	6
Medical Device and Object	Medical Device, Manufactured Object	2
Observation	Finding, Sign or Symptom, Injury or Poisoning, Laboratory or Test Result, Phenomenon or Process	5
Pathology	Disease or Syndrome, Neoplastic Process, Mental or Behavioral Dysfunction, Cell or Molecular Dysfunction, Pathologic Function	5
Physiology	Cell Function, Organ or Tissue Function, Organism Function*, Physiologic Function	4
Qualitative Concept	Qualitative Concept	1
Quantitative Concept	Quantitative Concept	1
Substance	Pharmacologic Substance, Substance, Biologically Active Substance, Biomedical or Dental Material*	4
Temporal Concept	Temporal Concept	1

We examined the top 100 mostly co-occurred relationships based on the weight of a relationship edge (total co-occurred incidences from the entire corpus) in the semantic network. We chose to include 100 top weighted relationships (e.g., “Location:Body Part”, “Observation:Body Part”) and 13 conceptually important relationships (e.g., “Substance: Observation”). Addition of 13 conceptually important relationships involved empirical input but it is essential to complement previous automatic entity extraction and analysis when generating a knowledge model. Subsequently, the proposed weight-based selection simplified the complex network by removing the co-occurred relationships with no obvious semantic relations, yet still revealed the structure of the sublanguage pattern.

To label the relationships, we selected 1000 “Subject/Object” instances within each of the 113 relationships in the knowledge model to make sure that all the relationships were covered. In addition, we made sure of at least five instances for each relationship. In total, we randomly selected 1000 “Subject/Object” instances from a pool of “Subject/Object” pairs generated from the 23,410 cases. Two physicians (JZ, Singapore General Hospital, Department of Internal Medicine; HZ, Greenfield Dental, Milwaukee, Wisconsin) were asked to assign specific relationship tags to each “Subject/Object” pair. The relationship tags were named explicitly based on the conceptual logic indicated by the “Relationship” (verb or preposition) in each SRO structure in a medical context; top examples are shown in Table 2. Later, we evaluated another 894 “Subject/Object” pairs from 83 randomly selected image reports.

**Table 2:** Ten most frequently co-occurred “Subject/Object” relationships identified from the corpus of 23,410 image reports

Co-occurrence Pair	Example	Count
Location:Body Part	frontal view:of(Situated_at):vertebral body; lower outer quadrant:of(Modifies):right breast	19,625
Observation:Body Part	erythema:of(Occurs_in):left breast; mass lesion(Occurs_in):in:left breast	15,219
Pathology:Body Part	B-cell lymphoma:of(Occurs_in):breast; fibroadenoma:with(Modifies):tissue	14,904
Medical Activity:Body Part	ultrasound: in(Acts_on):left breast; CT scans: of(Acts_on):skull	13,479
Observation:Pathology	x-ray findings:as(Indicative_of):pleural effusions; all features:of(Indicative_of):fibroadenoma	13,439
Functional Concept:Pathology	outcome:of(Describes):breast cancer; case:of(Related_to): previous DCIS	12,394
Pathology:Pathology	Haemangiomas:are(Be): benign vascular tumors; complications:include(Has):vessel thrombosis	12,119
Medical Activity:Pathology	drainage:confirmed(Shows):breast abscess; mastectomy:for(Acts_on):breast malignancy	11,924
Medical Activity:Observation	Chest x-ray:performed for(Deals_with):chest pain; biopsy:of(Acts_on):small lesion	11,890
Observation:Observation	breast lump:with(Shows):occasional pain; features:of(Shows):benign lesion	11,882

### Evaluation design

#### Knowledge model

The knowledge model was evaluated by using a corpus of 83 randomly selected image reports; including 43 image reports from Radiopaedia.org, 10 imaging case reports from the *Journal of Radiology Case Reports* [44], 15 case reports from the *BJR Case Report* [45], and 15 case reports from *RadioGraphics* [46]. Here we used data from four different sources in order to test the generalizability of our model, which was built from a single source. The corpus contained 402 sentence segments, 1718 noun phrases, and 894 “Subject/Object” pairs. Two independent raters with a clinical background (JZ and HZ) were asked to assign semantic annotations and relationship tags to each “Subject/Object” pair. Each rater received an instruction manual (see Additional file 1) that defined the scope and the meaning of induced semantic categories and the relationships among them. The manual also included examples to help raters assign semantic categories to identified noun phrases. The raters annotated the relationships in each SRO structure based on the manual; if they encountered any relationship not identified in the manual, they were asked to label the missed relationship with new category labels. We examined the raters’ tagging results and the default relationship tags offered by the formalized knowledge model. The relationship coverage is calculated as follows:

$$ \mathrm{Knowledge}\ \mathrm{model}\ \mathrm{relationship}\ \mathrm{coverage}=\frac{\#\mathrm{of}\ {\mathrm{raters}}^{\prime }\ \mathrm{tags}\ \mathrm{covered}\ \mathrm{by}\ \mathrm{the}\ \mathrm{knowledge}\ \mathrm{model}}{\mathrm{Total}\ \mathrm{Relationship}\ \mathrm{Counts}} $$

#### Machine annotation

For evaluation of machine annotation, currently, there is no gold standard to semantically model and evaluate radiology case reports. To generate a reference standard for evaluation, the 1676 noun phrases (excluding 42 noun phrases not covered by the knowledge model) were reviewed by two independent raters using the methods described in the previous section. On the other hand, the automatic machine annotation of semantic categories for the 1676 noun phrases was generated by the pipeline described previously. Later, the consensus results of the two raters were used as a reference standard to evaluate the machine annotations of semantic categories. Precision, recall, and F-score have been defined, respectively, as follows:

$$ \mathrm{Precision}=\frac{\mathrm{TP}}{\mathrm{TP}+\mathrm{FP}}; $$

$$ \mathrm{Recall}=\frac{\mathrm{TP}}{\mathrm{TP}+\mathrm{FN}}; $$

$$ \mathrm{F}-\mathrm{score}=2\ast \frac{\mathrm{Precision}\ast \mathrm{Recall}\ }{\mathrm{Precision}+\mathrm{Recall}\ } $$

The agreement was calculated by comparing the manual annotation of the raters. If the raters select the same label to annotate relationship, or same semantic category to annotate phrases, the annotation was considered as agreed. Otherwise, it was considered a disagreed annotation.

$$ \mathrm{Agreement}=\frac{\mathrm{Agreed}}{\mathrm{Agreed}+\mathrm{Disagreed}}. $$

## Results

### Semantic network analysis

**Fig. 3:**
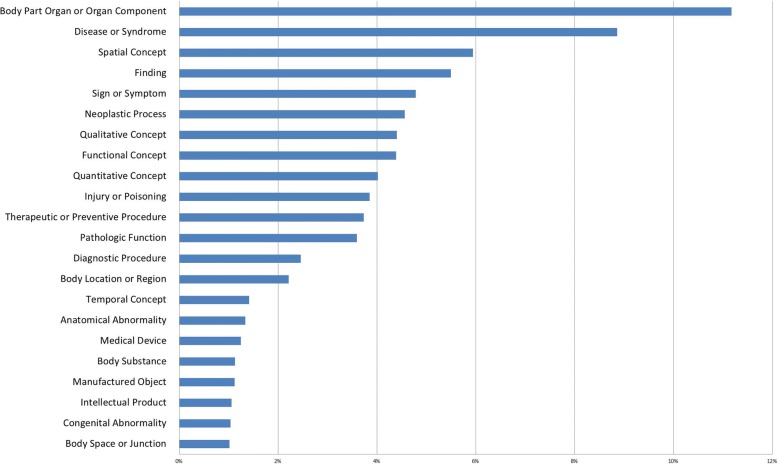
Summary of different semantic types (among 289,782 NP and ADJP, top 22). Majority (80.32%) of the radiology case corpus covered by the top 22 (16.3%) UMLS semantic types

The extracted semantic entities from the results of the syntactic processing stage included 289,782 noun phrases (NP) and adjective phrases (ADJP). The results of using 135 UMLS semantic types for semantic annotation demonstrated that the majority (80.32%) of the radiology cases in the corpus covered by the top 22 (16.3%) UMLS semantic types (Fig. 3). The resulting semantic network at this level was consisting of 135 nodes (semantic types) and 3492 distinct co-occurrence pairs, while 352,356 total co-occurrence incidences (each fall under 3492 distinct co-occurrence relationships) were extracted at the entity instance level.

We conducted a network analysis and extracted the top 100 important network relationships based on the weight (the number of co-occurrence incidences on the edges). This network indicated a strong sublanguage pattern among medical image reports, because (1) A small subset of semantic types was used to (top 40 + 4 expert chosen) cover a large amount of corpus (98%), and (2) there were many repeated relationships in the medical imaging reports’ entities. This led us to further generalize the semantic network into a knowledge model.

### Semantic type regrouping

To achieve high-quality semantic classification for entities [47] and to simplify the concept-relation representation [48], the semantic types in the network were regrouped into 14 semantic categories based on the hierarchical structure of UMLS [20, 49] and their position in the semantic network (Table 1). Among the 14 categories, five common UMLS types were reused without regrouping, including “Functional Concept”, “Qualitative Concept”, “Quantitative Concept”, “Temporal Concept”, and “Classification”. Regrouping the semantic types led to nine new semantic categories specific to image reports (Table 1). The top ten most frequent co-occurred “Subject/Object” relationships based on regrouped semantic types are shown in Table 2. The final knowledge model has 113 semantic relationships.

### Knowledge model

**Fig. 4:**
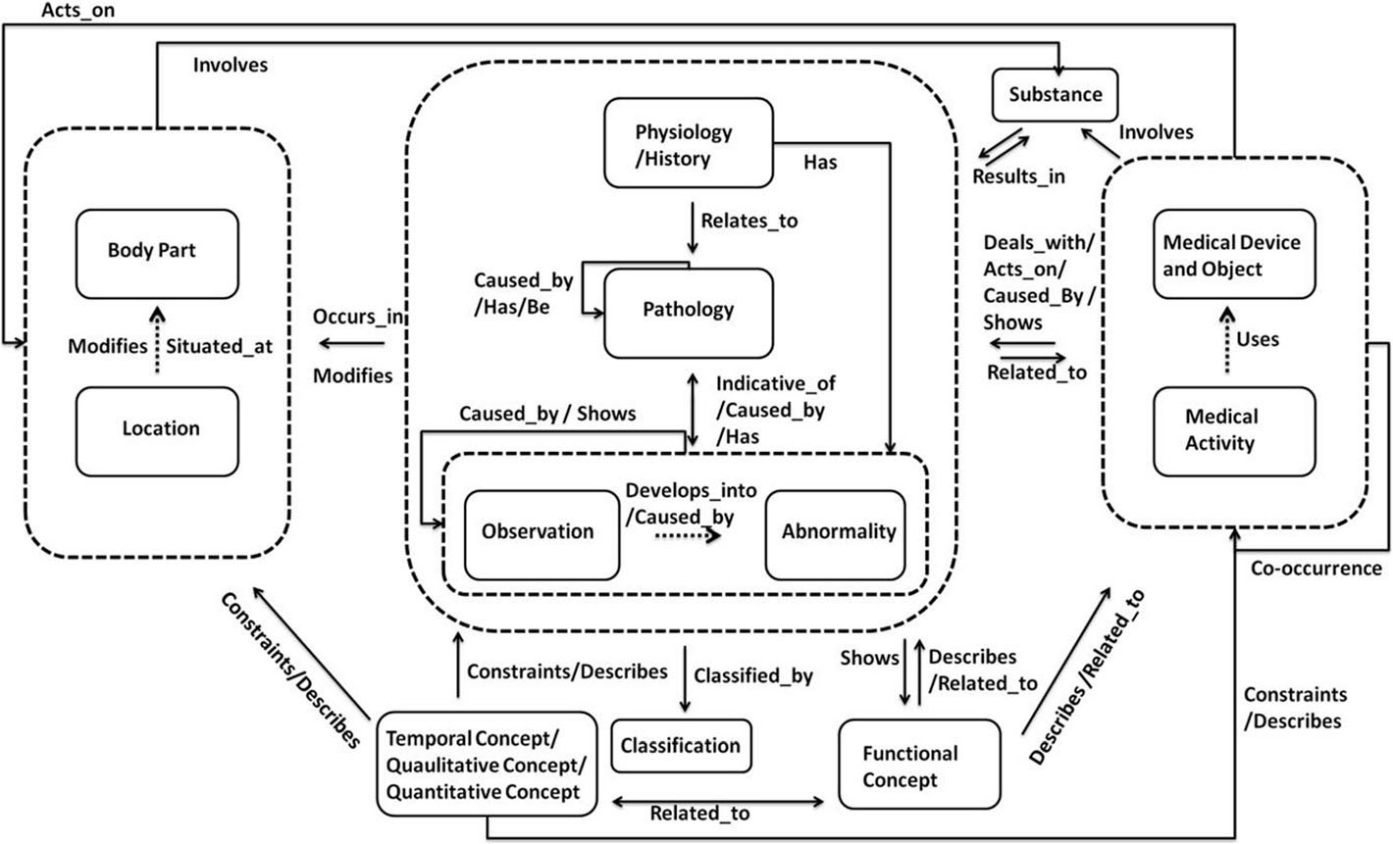
Knowledge model. The dotted lines show significant relationships in the co-occurrence network. The dotted box represents core semantic categories that are intrinsically closely related and are significant in the knowledge model

By linking the semantic categories with semantic relationships, we generalized a UMLS-based knowledge model for representing semantic information in medical image reports. The generated knowledge model is shown in Fig. 4; the significant relationships in the co-occurrence network are shown with the dotted lines, while the core semantic categories that are intrinsically closely related (determined by domain experts) and are significant in the knowledge model are presented in the dotted boxes. The significance of relationships and semantic categories were determined based on the total number of occurrence in the corpus.

**Fig. 5:**
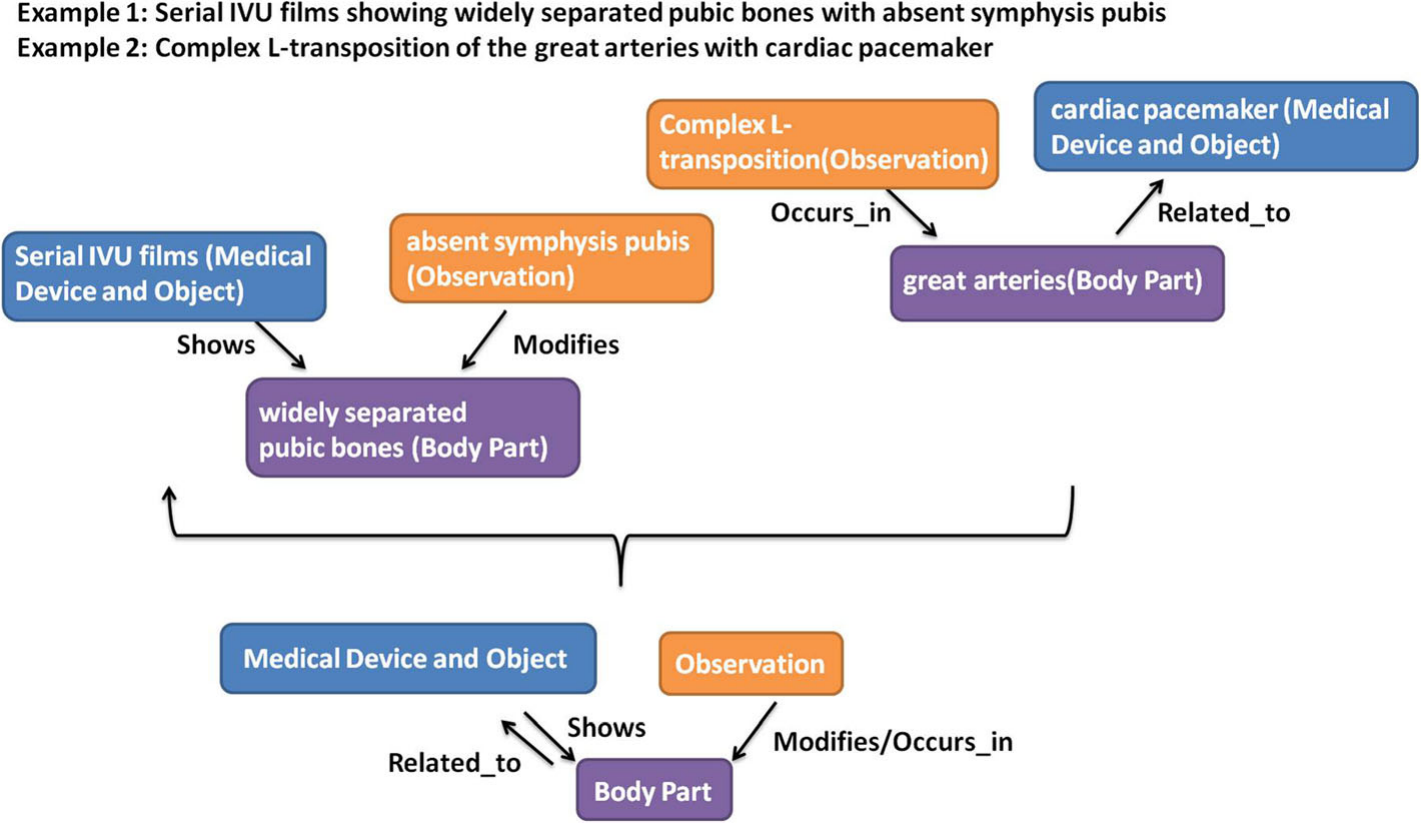
Knowledge model example of two sentences: “Serial IVU films showing widely separated pubic bones with absent symphysis” and “Complex L-transposition of the great arteries with cardiac pacemaker”

Figure 5 shows the semantic categories and relationships created for two sentences; “Serial IVU films showing widely separated pubic bones with absent symphysis” and “Complex L-transposition of the great arteries with cardiac pacemaker”. This image also shows how the created categories and relationships contribute to the generation of sub-sections of the overall knowledge model. The knowledge model provides a simple yet expressive view of content in the image reports, which can be used to facilitate future information retrieval and knowledge representation of medical image reports.

### Coverage evaluation of knowledge model

The initial inter-rater agreement was 92% for semantic annotation and 95% for relationship tags. After the raters’ discussion, the agreement reached 100%. The results showed that the use of 14 knowledge model semantic categories led into representing the semantics of 98% of the NP/ADJP, while 113 knowledge model relationships were required for annotation of 97% of the Subject/Object pair relationships. Additionally, 3% of the uncovered relationships involved some rare semantic types outside of the 14 semantic categories, such as “Biomedical Occupation or Discipline” and “Organism”.

### Evaluation of machine annotation

Based on our evaluation, machine annotation achieved an overall precision of 87%, recall of 79%, and F-score of 0.8299 (detailed evaluation results are listed in Table 3). Error analysis will be provided in the Discussion section.

**Table 3:** Evaluation of semantic annotation performance

Semantic Categories	True Positive (TP)	True Negative (TN)	False Positive (FP)	False Negative (FN)	Precision	Recall	F-Score
Abnormality	16	1660	4	12	80.0%	57.1%	0.6667
Body Part	238	1438	38	26	86.2%	90.2%	0.8815
Classification	12	1664	0	6	100.0%	66.7%	0.8000
Functional Concept	90	1586	14	12	86.5%	88.2%	0.8738
Location	230	1446	54	58	81.0%	79.9%	0.8042
Medical Activity	22	1654	14	26	61.1%	45.8%	0.5238
Medical Device and Object	8	1668	14	0	36.4%	100.0%	0.5333
Observation	168	1508	16	62	91.3%	73.0%	0.8116
Pathology	202	1474	4	50	98.1%	80.2%	0.8821
Physiology	16	1660	4	4	80.0%	80.0%	0.8000
Qualitative Concept	172	1504	22	60	88.7%	74.1%	0.8075
Quantitative Concept	78	1598	4	4	95.1%	95.1%	0.9512
Substance	24	1652	12	24	66.7%	50.0%	0.5714
Temporal Concept	56	1620	2	0	96.6%	100.0%	0.9825
Overall	1332	22,132	202	344	86.8%	79.5%	0.8299

## Discussion

In the medical domain, there are many complex relationships between entities, such as a clinical observation related to a certain pathology, or an observed disease co-occur with its comorbidities; therefore, we need a comprehensive knowledge model to support structured formalization of medical knowledge. A knowledge model (also referred to as an information model), is an important prerequisite for extracting information. The model has two components: (1) Semantic annotations that conceptualize entities in the imaging notes, and (2) relationships that link the discrete entities to form a logi/cal and integrated model. The advantage of our method, which extracts information based on the knowledge model, is discussed in the following sections. We also discuss the advantages of using semantic pattern mining to generate a knowledge model as follows;

### Compared to frame-based method for building knowledge model

Compared with previous studies that combined syntactic and semantic analysis and a pre-defined topic frame or event template to model information in a corpus [50–52], our knowledge model is able to provide a higher coverage of both semantic categories annotated and semantic relationships involved. In Friedman’s work [51], NPs were parsed into entities of problem and modifier (location, observation). For example, “Status post myocardial infarction” was framed as [problem, myocardial infarction, [status, post]]. Modifiers were generated around the core of the noun phrases “problem, myocardial infarction”. This approach had a limited scope since it was only able to distinguish the modifiers into “location” and “observations”. Here we didn’t compare our result directly with the Friedman study because 1) Friedman’s study did not report the coverage but only reported precision, recall, specificity, and sensitivity; 2) even though we also evaluated machine annotation performance using precision and recall, it is difficult to compare our task with previous studies since their tasks were disease specific and domain specific; 3) most frame-based templates were manually drafted, making it less likely to represent the true characteristics of a corpus for a specific domain. Our approach contributes to a data-driven and content-based perspective for generating knowledge model. The data-driven and content-based method is able to produce a knowledge model with higher coverage and more domain-specific representation. Thus, our knowledge model was able to cover 98% of the content in image notes corpus and reveal 97% of the relationships.

### Compared to machine learning-based method for building knowledge model

Several studies have explored the extraction of semantic relationships between entities using machine learning methods [53, 54]. Nevertheless, both methods require knowledge models to guide information extraction. For example, when training machine-learning algorithms (e.g., conditional random fields, SVM) to extract entities and their relationships in free-text, we first need to define a target model (e.g., entity labels, schema) to support machine annotation and relationship mapping. Previous studies often used knowledge models that were manually defined by experts focusing only on a specific domain, such as mammography and chest radiographic reports [55, 56]. By using a semantic network, we employed a novel approach that combines syntactic analysis with data-driven network analysis to explore semantic relations in a specific corpus. Compared with prior works that mostly involved syntactic analysis plus a rule-based or a supervised learning method to generate topic frames, our approach could potentially adapt to another corpus with reduced manual efforts.

### Compared to ontology-based method for building knowledge model

RadMiner [57] uses ontologies to represent the relationships between semantic entities. It can semantically analyze radiology reports using a clinical terminology called *Lexicon of Standardized Radiological Terms* (RadLex) [58]; however, concepts in the ontology model have complex relationships which are usually not well represented in the ontology itself. By a using context-based semantic network, we could better represent (higher coverage of) relationships between entities compared with other methods. By using UMLS, we also developed a knowledge model with a higher coverage than RadMiner, which uses RadLex.

RadMiner supports structured reporting of image findings and indexing of teaching cases. Despite its high coverage of anatomical structures, one study [59] showed that only 2.32% of phrases in a de-identified radiology report were exactly mapped to RadLex, while 50.53% of phrases were only partially mapped; in contrast, 10.40 and 85.95% of phrases were exactly and partially mapped to UMLS. Another study [60] demonstrated the lower coverage of RadLex for representing clinical language in imaging reports, especially for disease condition and non-radiology procedures; however, disease condition and non-radiology procedures comprise a significant percentage of content in image reports and case reports. Compared with RadMiner, our work provided a higher level and more comprehensive knowledge model comprising 14 semantic categories. We regrouped the most frequent UMLS semantic types into 14 semantic categories to reduce complexity results from the UMLS hierarchy or radiology language while still achieving a high coverage of radiology content.

### Subject:Relationship:Object structure

One advantage of using the SRO structure is that it can retain the relationships at the phrase level and reveal only the closest semantic relation in one sentence, thereby significantly reducing the chance for misinterpretation (“noises”). For example, if we analyze the sentence “There are foci of intensely increased radiotracer uptake in T9” at a sentence level, we will generate six co-occurrence relationships: “There/ foci, There/ intensely increased radiotracer uptake, There/T9, foci/T9, foci/ intensely increased radiotracer uptake, intensely increased radiotracer uptake/T9”. In contrast, if we analyze the sentence with the SRO structure, we will generate three relationships: “There:are:foci”, “foci:of: intensely increased radiotracer uptake in T9”, “intensely increased radiotracer uptake:in: T9”. These three relationships and their corresponding Subject and Object can be represented concisely.

### Content-based semantic type regrouping

We are aware of the complexity of UMLS hierarchical structure. Some recent studies have focused on reducing the complexity of radiology report content from an ontology perspective [21, 61, 62]. A pilot study [61] investigated the possibility of using 19 different vocabulary sources in UMLS to index XML-structured image reports. This study confirmed the enhancement of indexing precision of radiology reports by choosing the optimal subsets of UMLS vocabularies. In order to achieve high-quality semantic classification [47] and simplify concept relation representation [48], we regrouped the 40 most frequently occurring semantic types in our corpus into 14 major semantic categories. One of our main contributions in this work was a new regrouping strategy that incorporated a method, previously proposed by McCray et al. [20], and our domain specific adaptation. McCray’s method aggregated UMLS semantic types based on the inherent structure of UMLS. Our domain specific adaptation was based on the structure of the semantic network (Fig. 3).

### Clinically relevant granularity of noun phrases

Another novelty of our method was that we parsed maximal NP/ADJPs instead of base NP/ADJPs. Mapping entities according to base NP/ADJPs would result in returning a large amount of false positive results due to unsuitable granularity level. Our method, by keeping noun phrases intact and examining maximal NP/ADJPs instead of splitting one long NP/ADJPs into base NP/ADJPs and modifiers, was able to be regarded as a phrase-level information retrieval tool that filled the gap between word-level information retrieval (most of the prior work) and sentence-level information retrieval. Our method provided an efficient tool for tasks that would favor minimal query input but need a broader scope for information retrieval.

### Error analysis

Based on our evaluation results, we concluded that there would be five major causes for errors with machine annotation.

1. Some of the errors were caused by considering the tag of the last noun as the semantic type for the whole noun phrase. For example, “absent symphysis pubis” was considered “Observation” based on the examples in the annotation manual; however, as “symphysis pubis” was tagged as “Location”, it was then considered to be a “Location” concept instead of “Observation”.
2. Ambiguity in the meaning of words in a medical imaging context caused incorrect classification for UMLS semantic types. For example, “defect” was tagged as “Functional Concept” by the UMLS tagger, but actually, it is closer to an “Abnormality” in this context. In fact, the UMLS is known to associate numerous concepts with questionable semantic types.
3. Annotation error might also be caused by using a UMLS tagger trained on a general EHR corpus instead a more confined domain of medical image reports [41].
4. UMLS didn’t recognize typological errors and abbreviations. The low precision in “Medical Activity” was mostly caused by this type of error. For example “MRI TOF” was tagged as “MRI[Medical Activity] TOF[Abnormality]” instead of “MRI[Medical Activity] TOF[Medical Activity]”, because UMLS was not able to recognize the abbreviation of “TOF” as a Medical Activity.
5. Parsing error contributed to our overall error rate. Even though Stanford parser assumed to be less dependent on training corpus [63], it was shown previously that changing the word frequencies according to the medical context in the training corpus would improve parsing performance [64].

### Limitations and future work

One limitation of our work was that the relationships in the network were manually reviewed and labeled. Since our work mainly focused on the pipeline for generating a knowledge model, automatic relationship labeling was beyond our scope. However, it will be an interesting work for the future. In the future, we may be able to develop an annotated corpus based on our existing annotation of semantic entities and relationships, and then build an automated system to annotate relationships in image reports domain.

Another limitation is that our pipeline is not currently deployed in any framework (e.g. UIMA). Nor is it packaged into an installable software. However, since we have listed all the components of this work as a step-by-step diagram and have mentioned external software or packages we used in each step, the pipeline can be reproduced.

Other limitations come from our utilizing existing tools for parsing and annotating corpus. The tools are not trained on our specific domain, which may result in errors, as mentioned in the “Error Analysis” section. To reduce parsing errors, our future work will include retraining the parser and tailoring to the medical imaging domain. To solve the problems with incorrect semantic annotation, we can consider two approaches for future improvement: (1) Incorporate RadLex and FMA [65], which provides better semantic type assignment over Body Part, or incorporate other ontologies that have more comprehensive terminologies in “Medical Activity” and “Substance”, two low-performing UMLS semantic categories. (2) Reexamine and correct semantic types assignment errors based on specific domain context and avoid issues brought up by ambiguous and ill-defined UMLS semantic types, such as Functional Concept. (3) Future work to reduce errors caused by abbreviations or medical metaphors includes incorporating a list of common abbreviations/metaphors used in a radiology setting during the data processing step and adding spell-check modules to ensure better tagging quality.

At this time we cannot evaluate the precision and recall for the relationships, because we do not have an automated machine annotation for the semantic relationships; we can only automate the semantic annotation for the entities. The next step in our work is to create a machine annotation method for semantic relationships between the entities.

## Conclusions

We proposed a data-driven approach that used NLP and semantic network analysis to construct a knowledge model. We used medical image domain as a use case to demonstrate our system. The resulting knowledge model of medical image reports included 14 semantic categories and 113 semantic relationships. The evaluation using medical image reports from four different sources showed that the knowledge model created using a single source, Radiopaedia.org, was generalizable. The machine-tagging evaluation of 1676 entities achieved an overall precision of 87%, recall of 79%, and F-score of 82%. The knowledge model was able to cover 98% of the content in the evaluation corpus and revealed 97% of the relationships. This indicates that our knowledge model is comprehensive and covers a majority of concepts and relationships in medical image reports. Our pipeline to develop knowledge models demonstrated great potential of facilitating and improving information retrieval.

## Additional file

Additional file 1: Annotation manua. Annotation manual includes definitions of 14 semantic categories. It was used for evaluation of knowledge model. (XLSX 22 kb)

## Abbreviations

ADJP: Adjective Phrases
CDM: Common Data Model
EHR: Electronic Health Records
FMA: Foundational Model of Anatomy
NLP: Natural Language Processing
NP: Noun Phrases
PAS: Predicate-argument structures
PP: Prepositional Phrases
RadLex: Lexicon of Standardized Radiological Terms
RDF: Resource Description Framework
SRO: Subject:Relationship:Object
SVM: Support Vector Machines
UMLS: Unified Medical Language System
VP: Verb Phrases
